# Design of Jetty Piles Using Artificial Neural Networks

**DOI:** 10.1155/2014/405401

**Published:** 2014-08-07

**Authors:** Yongjei Lee, Sungchil Lee, Hun-Kyun Bae

**Affiliations:** ^1^Port and Harbor Team, Seoyeong Engineering, Republic of Korea; ^2^Department of Computer Design, School of Engineering and Agriculture, Ulaanbaatar University, Mongolia; ^3^Department of Global Environment, School of Environment, Keimyung University, 203 Osan Hall, Dalgubul-Daero, Dalsegu, Daegu 1095, Republic of Korea

## Abstract

To overcome the complication of jetty pile design process, artificial neural networks (ANN) are adopted. To generate the training samples for training ANN, finite element (FE) analysis was performed 50 times for 50 different design cases. The trained ANN was verified with another FE analysis case and then used as a structural analyzer. The multilayer neural network (MBPNN) with two hidden layers was used for ANN. The framework of MBPNN was defined as the input with the lateral forces on the jetty structure and the type of piles and the output with the stress ratio of the piles. The results from the MBPNN agree well with those from FE analysis. Particularly for more complex modes with hundreds of different design cases, the MBPNN would possibly substitute parametric studies with FE analysis saving design time and cost.

## 1. Introduction

Mooring dolphins are usually constructed when it would be impractical to extend the shore to provide access points to moor vessels. A typical mooring dolphin consists of a platform and several piles supporting the platform, which is so-called jetty. The vertical or battered piles are driven into the seabed. In design practice, deciding whether and where to use the vertical or battered piles is important issue. In the practical design process, the arrangement, the number, and the inclination of the piles are tentatively decided based on previous design experiences and then confirmed through finite element (FE) analysis. Therefore, building and analyzing lots of FE models adopting trial and error process are needed to find the optimum design.

Many researches have been performed to help designers to make decisions. An experimental study showed that the pile group effect is an important factor to resist horizontal loads [1]. For cyclic lateral loading, a zigzag arrangement shows higher resistance than an in-line arrangement. Also it was shown that as the pile center distance increases, the stresses on the front piles decrease, while those on the rear piles increase [2]. When the center distance between piles becomes more than 3~5 times of pile diameter, the group effect decreases so that each pile can be considered as a single pile when the distance reaches 6 times the pile diameter [3]. The battered piles are commonly considered to resist lateral loads solely while the vertical piles resist gravity loads only. This traditional design assumption would make the design process easier but also it usually results in overestimated design. Moreover, it is well known that the vertical piles can also resist bending moments from the lateral loads. Through empirical studies, the p-y method has been proposed and developed by Kondner [4], Reese et al. [5], Scott [6], and Norris [7] to help the design of jetty structure. Though it is still a commonly adopted method, some concepts of the method are based on oversimplified or improper assumptions, especially in the effects of actual soil parameters after pile driving [8].

To overcome the complication of jetty pile design originated from mutual interaction among a number of design parameters, artificial neural networks (ANN) have been introduced in geotechnical engineering [9, 10]. This technique has also been applied successfully in static and dynamic pile systems [11, 12]. Kim et al. [8] predicted the lateral behavior of single and group piles using ANN and compared the results from ANN with the model test results. In this paper, as a suggestive solution of difficulties and cumbersome processes in building and analyzing lots of FE models, the ANN is adopted. Possibility of substituting ANN as a jetty structure analyzer for FE analysis is examined.

## 2. Methodology: Application of ANN as a Structure Analyzer

**Figure 1:**
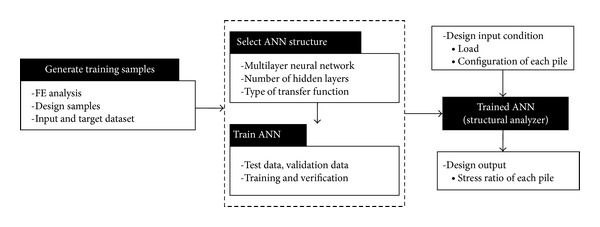
Methodology of design process using ANN.

The jetty design process involves, as mentioned above, searching for the optimum pile pattern which results in the most effective pile usage within feasible design region. The internal forces of the piles of jetty structure subjected to horizontal mooring load vary unexpectedly depending on the inclination of the piles and deployment pattern of piles. Therefore, developing ANN, the input data to ANN are decided as horizontal load exerting on the jetty platform and the information of jetty piles, such as arrangement and inclination of piles, and the output results as the stress ratios of piles to confirm the feasibility of design candidate. Whole concept of methodology adopted in this paper is summarized in Figure 1. As shown in Figure 1, the trained ANN is used as a structural analyzer in this research, placing FE analysis. Firstly, the training samples are generated using FE analysis for various design conditions. It is important that the training samples should be generated from various available design conditions so that the trained ANN may predict adequately when it encounters real new design data. Also the number of training samples should be large enough to avoid overfitting. In this research, total of fifty design cases with different loading conditions and pile patterns are considered for generating training samples through FE analysis.

**Figure 2:**
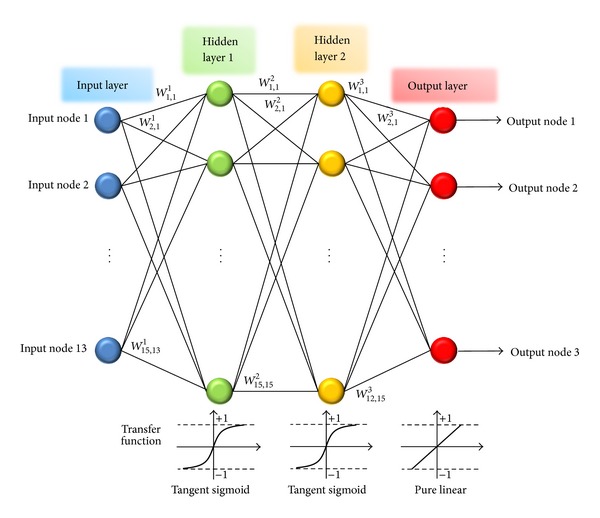
Neural network structure.

To construct the ANN architecture with predefined input and output layers, type of ANN, the number of hidden layers and neurons in each hidden layer, and type of transfer function for each layer should be determined. So in this research, because of the complexity of the problem, multilayer back-propagation neural network (MBPNN) with two hidden layers, shown in Figure 2, is adopted to tackle the problem. For the transfer function tangent sigmoid function and pure linear function are adopted for hidden layers and output layer, respectively, since the stress ratio, output from MBPNN, could be either compressive or tensional value. Each neuron of the hidden and output layers has bias and the neurons in one layer are interconnected with the neurons before and after the layer through weights.

Though the number of hidden layers of the MBPNN is determined as two, the performance of the MBPNN will vary depending on the number of neurons in hidden layers. Regarding the number of neurons in each hidden layer, however, there is no general rule to determine. Thus, in this study several neural network architectures with different number of neurons are examined for the best performance and generalization to new data based on the K-fold cross-validation method [13–15]. The performance and generalization of the MBPNN are summarized as an average of root mean squared error (RMSE) from the K-fold cross-validation. After fixing the architecture of MBPNN, training process is conducted to find the optimum values of the biases and weights using all fifty training samples. Finally, the trained MBPNN is used as a structural analyzer to produce the stress ratio of each pile for real design conditions.

## 3. Description of Jetty Structure for Analysis

**Figure 3:**
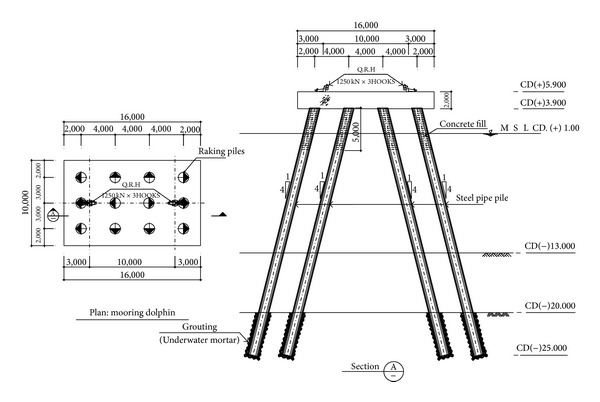
Mooring dolphin for U-project.

In this study, a mooring dolphin which was designed for a real project is used. Variations of the pile layout which had been proposed from the early design stage were also considered. The main purpose of the project was to design and build a liquefied natural gas (LNG) terminal at a port area so that the gas product would be transmitted from floating storage and regasification unit (FSRU) to natural gas network onshore by pipelines. The dimension of the platform is 16 m in length, 10 m in width, and 2 m in thickness. The platform is made of reinforced concrete and its piles are made of steel. The plan and elevation view of the testbed mooring dolphin are shown in Figure 3.

### 3.1. Materials

The material properties of C35/45 concrete for the platform are shown in Table 1. In the latest European standard BS EN 206-1 [16], the strength classes are classified using cylinder strength as well as a cube strength. S355 European standard steel is used for most of the structural members [17]. The material properties of S355 steel are shown in Table 2.

### 3.2. Load Conditions

The expected largest FSRU at the mooring dolphin has a capacity of 266,000 m^3^ and the largest LNG carrier has a capacity of 177,400 m^3^. Maximum mooring force is calculated as 3750 kN. Dead loads are listed in Table 3. All permanent structural members as well as nonstructural members have been considered as dead loads on the structure. Nonstructural member (appurtenance) includes quick release hook (QRH), fender, handrail, and grating. Detail appurtenance loads are shown in Table 4. Pedestrian live load of 4.0 kN/m^2^ is assumed. The maximum wave height varies between 2.5 and 2.75 m during a year, but about 60% days of a year wave height is less than 0.5 m. The mean (*T*_*m*−1,0_) wave period varies between 2.5 sec and 7 sec. Measurements about 4 km offshore indicate that the typical astronomical velocities are in the order of 0.5 m/s. The largest sea water current speed is 0.7 m/sec at the project site. The wind speed at the location is considered as 18 m/s. The maximum wind speed with 100 years of return period is 32.2 m/sec.

For the load combination, BS6349-2 [18] is adopted as shown:

(1) $$\begin{array}{c} \underset{j\geq 1}{\sum }\gamma_{G,j}G_{k,j}+\gamma_{p}P+\gamma_{Q,1}Q_{k,1}+\underset{j\geq 1}{\sum }\gamma_{Q,i}\psi_{0,i}Q_{k}, \end{array}$$

where *γ*_*G*_, *γ*_*Q*_ are partial factors, *P* is prestressing, *Q* is leading variable action, and *ψ*_0_ is combination factor.

### 3.3. Soil Conditions

The location of the virtual fixity points was computed by various methods: Chang's method, AASHTO, Hansen's method, and L-pile method. The pile penetration depth under the maximum tensile force was also computed by the Japanese bridge construction standard (2002), API recommended practice 2A-WSD, AASHTO, and Broms' analysis method. Based on those methods, it turned out that the penetration depth of 5 m into the bedrock would provide a fixed boundary condition at the bedrock level.

### 3.4. Design Configuration of Jetty Pile Pattern

**Figure 4:**
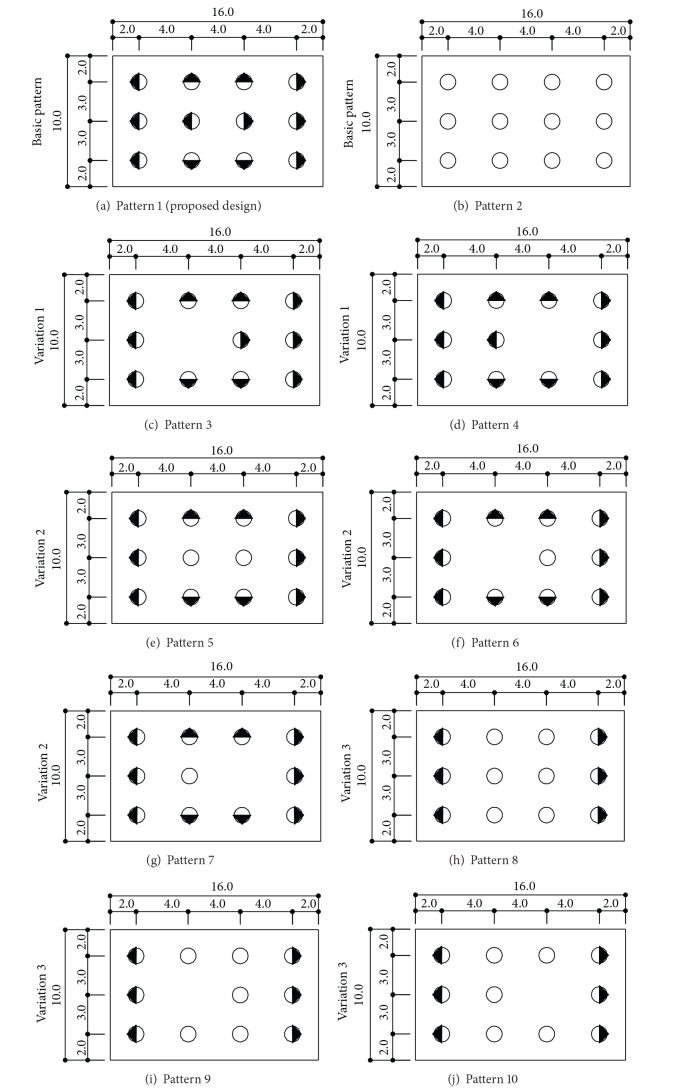
Patterns of piles (circle means vertical pile and circle with triangle means battered pile).

**Figure 5:**
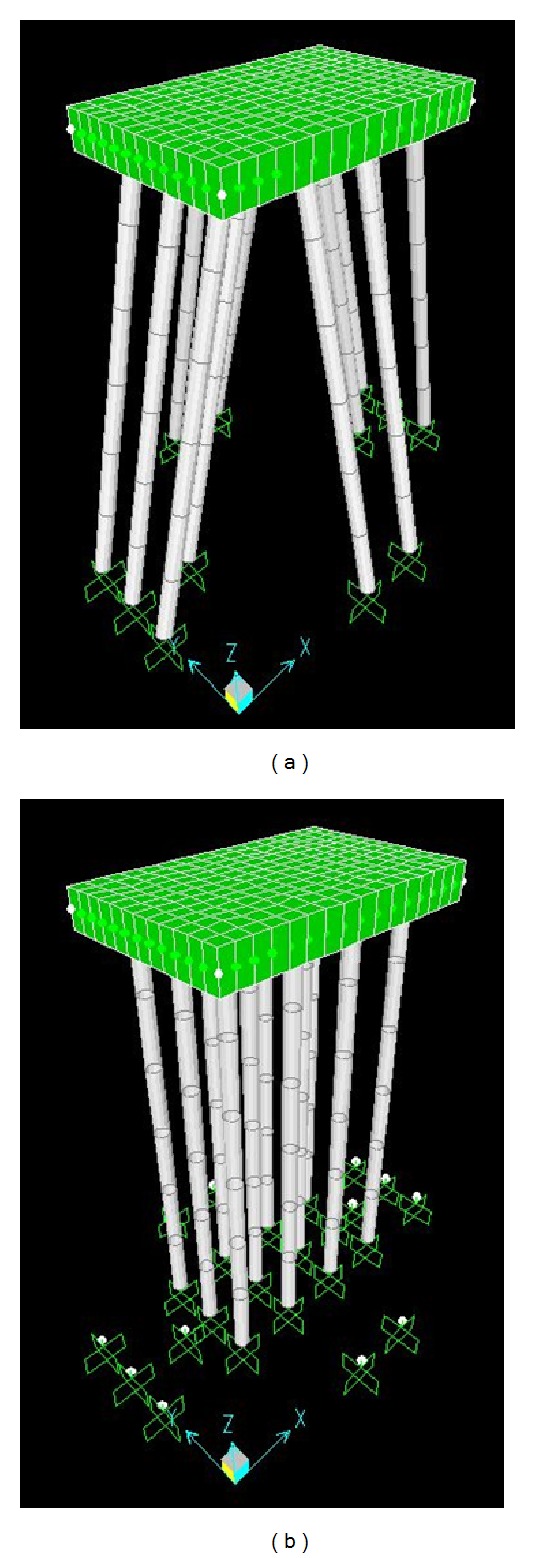
Finite element modes of Pattern 1 and Pattern 2.

Ten different configurations of jetty pile pattern are considered in this study depending on whether vertical or battered, if inclined, the batter direction, and the number of piles (Figure 4). The combination of ten different configurations and five different mooring forces (70%, 80%, 90%, 100%, and 110% of original mooring force) produced 50 FE models and they were analyzed to compute the stress ratios of piles. Among FE models, Patterns 1 and 2 are shown in Figure 5.

## 4. Preliminary FE Analysis

**Figure 6:**
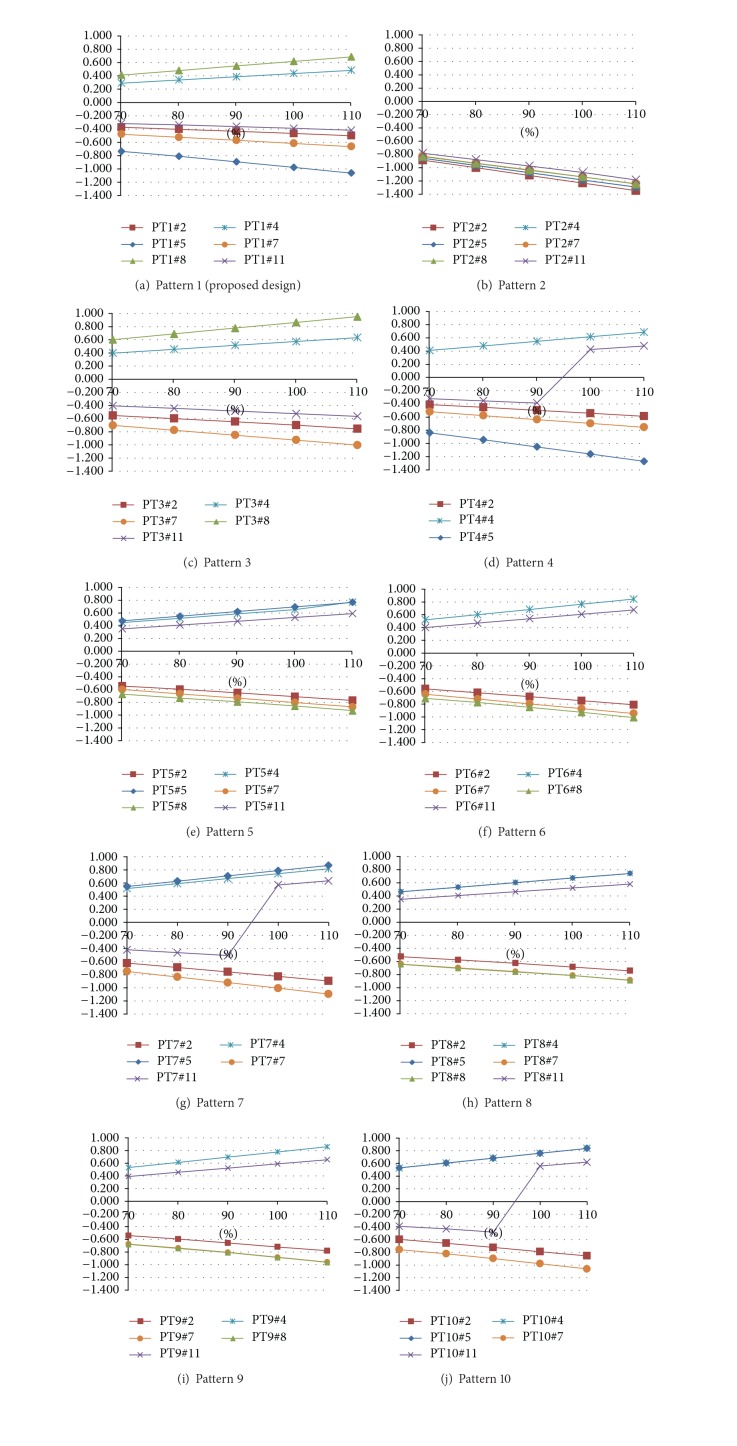
Maximum stress ratio of each pile.

After analyzing 50 FE models of mooring dolphins, the ratios of maximum stress to allowable stress of each pile were obtained under given loading condition as shown in Figure 6. Here, a pile pattern with the less number of required piles as well as with the smaller stress ratio is considered as the improved.

### 4.1. Comparison of Battered and Vertical Piles

In Pattern 1, the absolute values of the stress ratio are all less than unity except Pile 5. Pile 5 shows the maximum compressive stress and Pile 8 shows the maximum tensile stress. Pattern 2, whose piles are all vertical, shows compressive stress and most of the stress ratios are greater than unity.

### 4.2. Patterns 3 and 4

When Pile 5 is absent (Pattern 3), the tensile stress of Pile 8 becomes bigger than that of the proposed design (Pattern 1). In Pattern 3, the compressive force on Pile 5 in Pattern 1 redistributes to the adjacent piles. Considering all stress ratios and the number of piles, Pattern 3 can be considered as more improved design than the proposed one. Pile 11 of Pattern 4 is compressive within 90% of applied force but it turns to be tensile when the mooring force is equal to or more than 100%. In this case, the absence of Pile 8 causes a rapid stress change in Pile 11 and design of reinforcing bars in concrete platform is difficult; therefore Pattern 4 shall be avoided.

### 4.3. Patterns 5, 6, and 7

When it is compared to Pattern 1, Pile 4 of Pattern 5 remains tensile. However, Piles 4 and 8 of Pattern 1 change to be compressive in Pattern 5, while Piles 5 and 11 become tensile. The use of vertical piles in Pattern 5 makes stress sign changed. All absolute values of stress ratio of Pattern 5 are less than unity. In this point of view, Pattern 5 is more effective than the proposed design. With absence of Pile 5, Pattern 6 shows slightly higher stress than Pattern 5. Similar to Pattern 4, the absence of Pile 8 causes rapid stress change in Pile 11 of Pattern 7. Pattern 7 shows higher compressive stress than Pattern 5.

### 4.4. Patterns 8, 9, and 10

The stress ratios of Patterns 8, 9, and 10 are similar to those of Patterns 5, 6, and 7 but they are not compatible for horizontal load direction change.

## 5. Architecture and Training of MBPNN

### 5.1. Training Samples

The first neuron of the input layer is assigned for load condition, and the other input neurons take the information of piles. To distinguish the battered and the vertical piles, the numbers “1” and “2” are assigned as neuron input values for each pile. To the location where pile is absent, the number “0” is assigned to the corresponding input neuron. For the value of mooring force corresponding to the value of input neuron 1 which is very big compared with the values of the other input neurons might lead to a failure of MBPNN training, the mooring force is normalized to the mooring force of 3750 kN.  Table 5 shows the example of input values and corresponding pile locations.

In this study, MBPNN, with two hidden layers, utilizing back-propagation process is used. To obtain the training samples, five load cases—70%, 80%, 90%, 100%, and 110% of mooring force of 3750 kN—are applied to 10 different pile patterns of jetty structures. The combination of 10 jetty pile patterns and 5 load cases makes 50 training cases in total.  Table 6 shows an example of input and target output of training samples.

### 5.2. Construction of MBPNN Architecture and Training

Since the performance and generalization of MBPNN to new design data will vary depending on the number of neurons in hidden layers, four different topologies of MBPNN with different number of neurons in hidden layers are examined: (1) 13 (input neurons)-15(1st hidden layer)-15 (2nd hidden layer)-12 (output layers), (2) 13-10-10-12, (3) 13-7-15-12, and (4) 13-15-10-12. The number of neurons in hidden layers of the first MBPNN model (13-15-15-12) is greater than that of input or output layers and vice versa in the second model (13-10-10-12). In this paper, the K-fold cross-validation method is used to assess the generalization of model and to select the best architecture of MBPNN. For the K-fold cross-validation the fifty training samples are randomly divided into 10 subsets, that is, 10-fold cross-validation. In the K-fold cross-validation one subset is assigned as validation data set and the other nine subsets as training data set. Each MBPNN model is trained using the training data set and RMSE is computed for the validation data set. This procedure continues 10 times changing validation data set and training data set. Finally, a MBPNN model with the least averaged RMSE is selected as the best model.

The neural network toolbox provided by commercial program MATLAB was used to construct and for training of MBPNN models. The Levenberg-Marquardt method with back-propagation process was adopted for the optimization algorithm for training. The Levenberg-Marquardt method is considered to be effective for the complicated MBPNN for the fastest training [19, 20]. The objective function in the neural network training is defined as the minimization of mean squared error as shown:

(2) $$\begin{array}{c} \text{Objective}\;\text{Function}=\text{Minimization}:\frac{1}{n}\overset{n}{\underset{i=1}{\sum }}{\left(y_{i}-f\left(x_{i},\delta \right)\right)}^{2}, \end{array}$$

where *n* = total number of training pattern, *y*_*i*_ = *i*th target, *x*_*i*_ = *i*th input, and *δ* = weights and biases of neural network.

In the Levenberg-Marquardt method, the optimum weights and biases are searched using

(3) $$\begin{array}{c} \delta_{k+1}=\delta_{k}-{\left(J^{T}J+\mu \mathrm{diag}\left(J^{T}J\right)\right)}^{-1}J^{T}e, \end{array}$$

where *δ*_*k*+1_ = (*k* + 1)th weights and biases, *J* = Jacobian matrix, *μ* = adaptive  value, and *e* = error.

The successful performance of the Levenberg-Marquardt method depends on the choice of *μ*. Where the gradient is small, the search movement should be large so that the slow convergence is avoided. However, it should be small for the steeper gradient region. The initial value of *μ* was assumed as 0.001, and the increase and decrease factor of *μ* were assigned as 10 and 0.1, respectively. Thus, during the training, *μ* will take the value of 0.001 × (increase  factor  or  decrease  factor)^*n*^, where *n* is zero or natural number. The training process starts with the initial value and at the second step the objective function (i.e., mean squared error) is computed with the previous value of *μ* = 0.001 and *μ* = 0.001 × (decrease  factor). If both of these values do not result in good performance, a new value of *μ* = 0.001 × (increase  factor) is adopted for the next step. In the following steps, the best *μ* value is searched among “previous *μ* value,” “(previous  *μ*  value)×(decrease  factor),” and “(previous  *μ*  value) × (increase  factor)^*n*^.” If a previous *μ* value results in reduction of the objective function, the value is not changed.

Table 7 summarizes the averaged RMSE of each MBPNN model from the K-fold cross-validation. Interestingly the first model (13-15-15-12) which is the most complex one among the four models does not show the least averaged RMSE, rather than the second model of which the number of neurons in hidden layers is between that of input and output layer which shows the least. From the K-fold cross-validation results, the second MBPNN model with 10 neurons for each hidden layer is selected as the best architecture.

**Figure 7:**
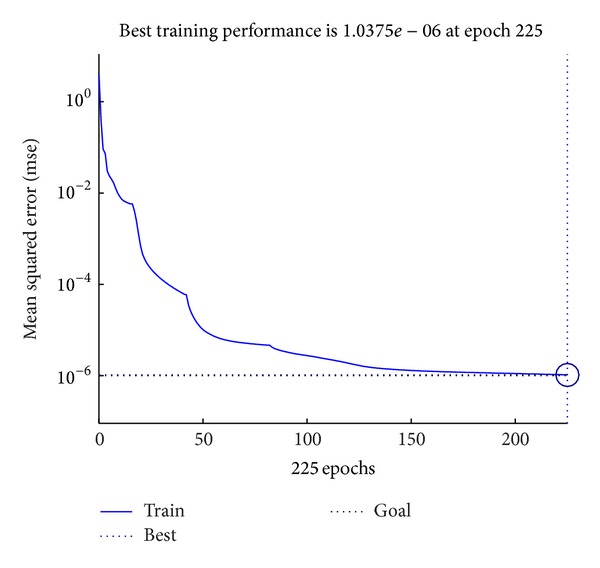
Training performance.

**Figure 8:**
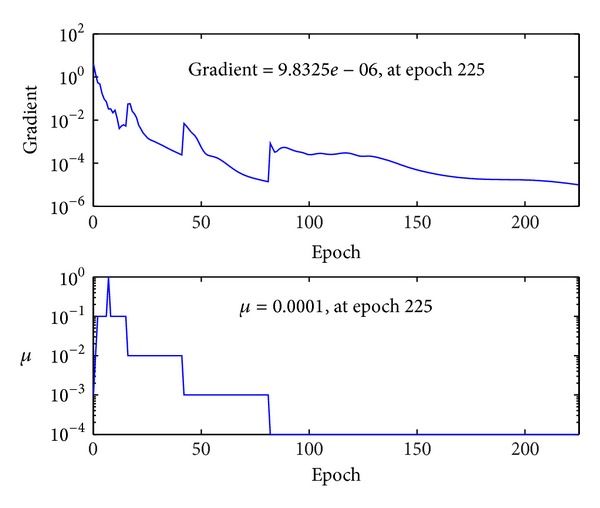
Change of gradient and *μ* at each training epoch.

After fixing the architecture of the MBPNN, the MBPNN was trained again with all the training samples. The training process was completed at 147 epochs, as shown in Figure 7, and terminated at performance goal of 10^−6^ which was set as one of the termination conditions.  Figure 8 shows the gradient changes of the problem surface and changes of the *μ* value during the training process. If a *μ* value gives the reduction of objective function, the value is kept for the following steps. However, if it does not result in good performance, it is updated using the increase or decrease factor. To find the optimum point, the *μ* value is continuously updated during the training process and with the change of the *μ* value the searching direction and gradient of problem surface are changed. As long as the gradient is large enough to improve the training performance, the *μ* value is unchanged; however, when the gradient gets smaller and the training performance gets worse, which means the surface of the objective function becomes flat, the *μ* value is updated. Comparing the training performance graph and the *μ* graph, it is observed that the *μ* value starts from the initial value of 0.001 and is updated at epochs 2, 7, 16, 42, and 82. In Figure 8 with the constant *μ* values the performance and gradient become flat; however, the training performance and gradient are improved dramatically at those epochs.

### 5.3. Verification of Training

Cases 3, 15, 27, 39, and 50 were selected for verification of MBPNN training.  Table 8 shows the comparison of 5 known targets of training cases and the simulated results from the trained MBPNN. The values in the last row are root mean squared error (RMSE) between the known targets and the simulated values. At the locations of no pile, the target value is 0 and the simulation results also show similar value and RMSE is very small. This ensures that the MBPNN is well trained.

## 6. Design with Trained MBPNN

Through the trained MBPNN, the stress ratios of jetty piles were obtained under different loading conditions which were not included in the training samples. The feasibility of the MBPNN was verified by comparing the results from FE model and the MBPNN.  Table 9 shows the stress ratios computed by FE analysis and the MBPNN. The results from the MBPNN are very close to the FE analysis results. The RMSE is also very small regardless of the pile patterns.

## 7. Conclusions

In this paper, the application of MBPNN as a structural analyzer for jetty structures is explored. The framework of MBPNN is defined as the input with the lateral forces on the jetty structure and the type of piles and the output with the stress ratios of the piles. For the highly complex jetty pile patterns the results from the MBPNN show very good agreement with those from FE analysis. With the more training samples and the expansion of input parameters for jetty structure design, the MBPNN shows possibility to replace the repetitive and time-consuming FE analysis. Although only 50 cases have been modeled for study purpose in this paper, the merit of MBPNN would be clearer as the number of cases increases.

## Figures and Tables

**Table 1:** Material properties of concrete.

Unit weight of concrete (dry/submerged)	kN/m^3^	23/13.19
Unit weight of RC (dry/submerged)	kN/m^3^	24/14.19
Cylinder strength of RC (*f*_cu_)	N/mm^2^	35
Cube strength of RC (*f*_cu_)	N/mm^2^	45
Modulus of elasticity (*E*_*c*_)	kN/mm^2^	29
Poisson's ratio (*ν*)		0.2

**Table 2:** Material properties of S355 steel.

Thick.	*t* ≤ 16	16 < *t* ≤ 25	25 < *t* ≤ 40	40 < *t* ≤ 63	63 < *t* ≤ 80	80 < *t* ≤ 100	100 < *t* ≤ 150
Yield strength (MPa)	355	345	345	335	325	315	295
Tensile strength (MPa)	460–620						
Modulus of elasticity (*E*)	205 kN/mm^2^						
Shear modulus (*G*)	80 kN/mm^2^						
Poisson's ratio (*ν*)	0.3						

**Table 3:** Dead load.

(ton/m^3^)	RC	Concrete	Mortar	Steel	Rubble	Fill sand	Sea water
Dry	2.45	2.30	2.15	7.85	0.8	2.0	1.025
Submerged	1.45	1.30	1.15	6.85	1.8	1.0	

**Table 4:** Appurtenance loads.

Nonstructural member	Loads	Remark
Q.R.H	49 kN/EA	Vertical load
Cone type fender (1800 H (F0.3) or equivalent)	196 kN/EA	
Handrail	0.285 kN/m	
Grating	0.838 kN/m^2^	

**Table 5:** Example of neuron inputs for neural network training process.

Input neuron		Remark
Number	Assigned value	
1	1.0	Normalized value of horizontal mooring force to 3750 kN
2	1.0	Pile 1: battered
3	1.0	Pile 2: battered
⋮	⋮	⋮
12	2.0	Pile 11: vertical
13	2.0	Pile 12: vertical

**(a):** 

Cases	Input neuron (load and information of each pile)								
	1 (Load)	2 (Pile 1)	3 (Pile 2)	4 (Pile 3)	5 (Pile 4)	*⋯*	11 (Pile 10)	12 (Pile 11)	13 (Pile 12)
1	0.7	1	1	1	1	*⋯*	1	1	1
2	0.7	2	2	2	2	*⋯*	2	2	2
3	0.7	1	1	1	1	*⋯*	1	1	1
*⋮*	⋮	⋮	⋮	⋮	⋮	*⋮*	⋮	⋮	⋮
48	1.1	1	1	1	2	*⋯*	1	1	1
49	1.1	1	1	2	0	*⋯*	1	1	1
50	1.1	1	1	1	2	*⋯*	1	1	1

**(b):** 

Cases	Output neuron (stress ratio of each pile)							
	1 (Pile 1)	2 (Pile 2)	3 (Pile 3)	4 (Pile 4)	5 (Pile 5)	*⋯*	11 (Pile 11)	12 (Pile 12)
1	−0.364	−0.363	−0.364	0.302	−0.727	*⋯*	−0.307	−0.305
2	−0.877	−0.877	−0.877	−0.847	−0.847	*⋯*	−0.771	−0.771
3	−0.547	−0.548	−0.547	0.404	0.404	*⋯*	−0.399	−0.397
⋮	⋮	⋮	⋮	⋮	⋮	⋮	⋮	⋮
48	−0.750	−0.751	−0.750	0.737	0.737	*⋯*	0.573	0.574
49	−0.788	−0.787	−0.788	0.856	0.856	*⋯*	0.651	0.651
50	−0.863	−0.864	−0.863	0.831	0.830	*⋯*	0.613	0.616

**Table 7:** RMSE of each MBPNN model from K-fold cross-validation.

Round	MBPNN model			
	13-15-15-12	13-10-10-12	13-7-15-12	13-15-10-12
1	0.0130	0.0986	0.0482	0.1205
2	0.0606	0.0125	0.0796	0.0118
3	0.0192	0.0086	0.1495	0.0238
4	0.0295	0.0046	0.0675	0.0264
5	0.1138	0.0986	0.1181	0.0078
6	0.1733	0.0383	0.1607	0.0413
7	0.0147	0.0346	0.1218	0.1053
8	0.1147	0.0643	0.0791	0.0730
9	0.1396	0.0461	0.1098	0.2451
10	0.0052	0.0258	0.0343	0.0131

Averaged	0.0684	0.0432	0.0969	0.0668

**Table 8:** Simulation results.

Pile number	Number of training cases									
	3		15		27		39		50	
	Target	Simul.	Target	Simul.	Target	Simul.	Target	Simul.	Target	Simul.
1	−0.6050	−0.6051	−0.6030	−0.6035	−0.7580	−0.7580	−0.7120	−0.7117	−0.7500	−0.7492
2	−0.6050	−0.6055	−0.6030	−0.6032	−0.7580	−0.7588	−0.7120	−0.7121	−0.7510	−0.7496
3	−0.6050	−0.6051	−0.6030	−0.6035	−0.7580	−0.7580	−0.7120	−0.7117	−0.7500	−0.7492
4	0.5230	0.5227	0.6090	0.6082	0.6680	0.6673	0.6540	0.6537	0.6400	0.6398
5	0.5220	0.5227	0^†^	0.0001	0.7080	0.7092	0.6940	0.6937	0^†^	−0.0004
6	0.5230	0.5227	0.6090	0.6082	0.6680	0.6672	0.6540	0.6538	0.6400	0.6399
7	−0.7660	−0.7649	−0.7440	−0.7433	−0.9210	−0.9210	−0.8060	−0.8057	−0.9990	−0.9990
8	0^†^	−0.0008	−0.7490	−0.7475	0^†^	−0.0004	−0.8600	−0.8599	0.9600	0.9593
9	−0.7660	−0.7657	−0.7440	−0.7432	−0.9210	−0.9203	−0.8060	−0.8059	−0.9990	−1.0010
10	−0.3970	−0.3963	0.4530	0.4528	−0.5100	−0.5096	0.5310	0.5302	−0.5580	−0.5580
11	−0.3980	−0.3974	0.4520	0.4521	−0.5080	−0.5093	0.5300	0.5294	−0.5600	−0.5600
12	−0.3970	−0.3963	0.4530	0.4528	−0.5100	−0.5096	0.5310	0.5302	−0.5580	−0.5580

RMSE	0.0008		0.0004		0.0007		0.0006		0.0005	

RMSE: root mean squared error.

^†^No pile at this location.

**Table 9:** Comparison of stress ratios obtained by MBPNN and FE analysis.

Pile number	Patterns (75% of original mooring force)									
	1		2		3		4		5	
	Anlys.	ANN	Anlys.	ANN	Anlys.	ANN	Anlys.	ANN	Anlys.	ANN
1	−0.3800	−0.3803	−0.9350	−0.9333	−0.5710	−0.5697	−0.4290	−0.4297	−0.5720	−0.5710
2	−0.3790	−0.3788	−0.9350	−0.9335	−0.5710	−0.5701	−0.4270	−0.4281	−0.5730	−0.5713
3	−0.3800	−0.3803	−0.9350	−0.9333	−0.5710	−0.5697	−0.4290	−0.4297	−0.5720	−0.5710
4	0.3260	0.3259	−0.9020	−0.9004	0.4330	0.4333	0.4500	0.4486	0.4830	0.4834
5	−0.7610	−0.7633	−0.9020	−0.9012	0^†^	−0.0004	−0.8870	−0.8873	0.5120	0.5127
6	0.3260	0.3261	−0.9020	−0.9004	0.4330	0.4333	0.4500	0.4487	0.4830	0.4834
7	−0.4910	−0.4885	−0.8690	−0.8679	−0.7360	−0.7347	−0.5450	−0.5442	−0.6350	−0.6357
8	0.4590	0.4580	−0.8740	−0.8727	0.6530	0.6529	0^†^	0.0005	−0.7050	−0.7038
9	−0.4910	−0.4790	−0.8690	−0.8678	−0.7360	−0.7357	−0.5450	−0.5437	−0.6350	−0.6346
10	−0.3140	−0.3154	−0.8190	−0.8147	−0.4170	−0.4168	−0.3320	−0.3308	0.3820	0.3825
11	−0.3160	−0.3171	−0.8190	−0.8146	−0.4190	−0.4187	−0.3320	−0.3313	0.3810	0.3814
12	−0.3140	−0.3154	−0.8190	−0.8147	−0.4170	−0.4168	−0.3320	−0.3308	0.3820	0.3825
RMSE	**0.0037**		**0.0025**		**0.0007**		**0.0010**		**0.0008**	

Pile number	Patterns (75% of original mooring force)									
	6		7		8		9		10	
	Anlys.	ANN	Anlys.	ANN	Anlys.	ANN	Anlys.	ANN	Anlys.	ANN

1	−0.5900	−0.5918	−0.6550	−0.6556	−0.5590	−0.5587	−0.5750	−0.5749	−0.6330	−0.6336
2	−0.5900	−0.5912	−0.6240	−0.6563	−0.5600	−0.5593	−0.5740	−0.5747	−0.6340	−0.6341
3	−0.5900	−0.5918	−0.6550	−0.6556	−0.5590	−0.5587	−0.5750	−0.5749	−0.6330	−0.6336
4	0.5610	0.5598	0.5540	0.5540	0.4920	0.4920	0.5680	0.5670	0.5620	0.5615
5	0^†^	−0.0001	0.5880	0.5869	0.4920	0.4921	0^†^	−0.0001	0.5610	0.5599
6	0.5610	0.5599	0.5540	0.5540	0.4920	0.4920	0.5680	0.5670	0.5620	0.5615
7	−0.6840	−0.6834	−0.7930	−0.7928	−0.6770	−0.6757	−0.7130	−0.7107	−0.7990	−0.7973
8	−0.7430	−0.7420	0^†^	−0.0003	−0.6830	−0.6825	−0.7180	−0.7158	0^†^	0.0003
9	−0.6840	−0.6833	−0.7930	−0.7917	−0.6770	−0.6750	−0.7130	−0.7106	−0.7990	−0.7981
10	0.4350	0.4353	−0.4410	−0.4396	0.3710	0.3718	0.4200	0.4200	−0.4170	−0.4161
11	0.4350	0.4344	−0.4420	−0.4398	0.3700	0.3705	0.4190	0.4192	−0.4180	−0.4169
12	0.4350	0.4353	−0.4410	−0.4396	0.3710	0.3718	0.4200	0.4200	−0.4170	−0.4161
RMSE	**0.0010**		**0.0094**		**0.0008**		**0.0013**		**0.0009**	

RMSE: root mean squared error.

^†^No pile at this location.
